# Effect of the Addition of Graphene Nanoplatelets on the Thermal Conductivity of Rocket Kerosene: A Molecular Dynamics Study

**DOI:** 10.3390/ma15165511

**Published:** 2022-08-11

**Authors:** Xiaodie Guo, Xuejiao Chen, Jinpeng Zhao, Wenjing Zhou, Jinjia Wei

**Affiliations:** 1 School of Chemical Engineering and Technology, Xi’an Jiaotong University, Xi’an 710049, China; 2 Beijing Institute of Aerospace Testing Technology, Beijing 100074, China; 3 State Key Laboratory of Multiphase Flow in Power Engineering, Xi’an Jiaotong University, Xi’an 710049, China

**Keywords:** rocket kerosene, graphene nanoplatelets, thermal conductivity, molecular dynamics

## Abstract

Rocket kerosene plays an important role in the regenerative cooling process of rocket thrust chambers. Its thermal conductivity determines the cooling efficiency and the tendency to coke in rocket kerosene engines. In this paper, graphene nanoplatelets (GNPs) were introduced into rocket kerosene to improve its thermal conductivity. Molecular dynamics simulation was used to investigate the thermal conductivity of the composite system and its underlying thermal conductivity mechanism. Firstly, by studying the effect of the mass fraction of GNPs, it was found that, when the graphene mass fraction increases from 1.14% to 6.49%, the thermal conductivity of the composite system increases from 4.26% to 17.83%, which can be explained by the percolation theory. Secondly, the influence of the size of GNPs on the thermal conductivity of the composite system was studied. Basically, the thermal conductivity was found to increase by increasing the aspect ratio of GNPs, indicating that GNPs with a higher aspect ratio are more conducive to improving the thermal conductivity of rocket kerosene. By carefully analyzing the effect of the size of GNPs on thermal conductivity, it was concluded that the thermal conduction enhancement by adding GNPs is determined by the combined effect of the percolation theory and the Brownian motion. The results of the temperature effect study showed that the ratio of thermal conductivity to rocket kerosene increased from 1.16 to 1.26 and from 1.07 to 1.11 for the composite systems, with graphene sizes of 41.18 Å × 64.00 Å and 24.14 Å × 17.22 Å in the temperature range of 293 K to 343 K, respectively. It is further proved that the Brownian motion of GNPs has a non-negligible effect on the thermal conductivity of the composite system. This work provides microscopic insights into the thermal conduction mechanism of GNPs in nanofluids and will offer practical guidance for improving the thermal conductivity of rocket kerosene.

## 1. Introduction

In the thrust chamber of a rocket engine, as the thrust increases, a high heat flux will be generated on the wall of the thrust chamber. In order to keep the temperature of the thrust chamber wall within the allowable range, it needs to be regeneratively cooled [1,2]. Before entering the combustion chamber, the rocket kerosene will be used as a coolant to regeneratively cool the thrust chamber wall. Thermal conductivity is an important factor to measure the cooling capacity of the liquid propellant. Kerosene with high thermal conductivity can not only effectively improve heat transfer efficiency but also prevent coking caused by high temperatures in the regenerative channel. Therefore, in consideration of the safety and reliability of rocket engine operation, it is necessary to increase the thermal conductivity of rocket kerosene.

Graphene has attracted much attention since it was discovered in 2004 because of its special physical and chemical properties. Compared with other unique properties of graphene, its novel thermal properties have been widely used in industry and academia [3,4,5]. Adding graphene nanoplatelets (GNPs) has been proven to be an effective way to improve the thermal conductivity of fluid [6]. Compared with other nanoparticles such as metals and some inorganic non-metallic materials, the thermal conductivity of graphene can reach 5300 W/m·K [7], which has made it a research hotspot in recent years. For example, Shi et al. [8] measured the thermal conductivity of graphene/paraffin composite in their experiments, and the results showed that the thermal conductivity of paraffin was 0.25 W/m·K, and that of the paraffin/graphene composite reached 0.5 W/m·K when 10 wt% graphene was loaded. Yavari et al. [9] investigated the thermal conductivity of graphene/1-octadecanol, and the results showed that the thermal conductivity of organic phase change materials could be significantly improved by adding graphene platelets. When 4 wt% graphene was added, the thermal conductivity of the nanocomposites increased by about 140%. The thermal conductivity enhancement effect was significantly better than that of other nano-fillers such as silver nanowires. Fang et al. [10] added graphene nanosheets of different mass fractions (0, 1, 2, 5, and 10 wt%) to eicosane and found that, when the mass fraction of graphene nanosheets was 10%, the thermal conductivity was increased by more than 400%.

Increased thermal conductivity in nanofluids has been widely observed, but how to explain this phenomenon remains controversial. In previous studies, a variety of mechanisms have been proposed to explain the increased thermal conductivity, such as: (1) the percolation theory of Nanoparticles [11,12,13], (2) the Brownian motion of nanoparticles [14,15], (3) the aggregation of nanoparticles [11,16,17], (4) liquid layering theory [11,18], and (5) Ballistic phonon heat transfer [11]. However, researchers have not reached a consensus on these mechanisms, and extensive research is needed on the thermal conductivity mechanism behind nanofluids.

Many researchers have pointed out that the shape and size of nanoparticles is an important factor affecting the thermal conductivity of nanofluids. For example, Das et al. [19] emphasized the importance of particle size, and it was considered that the stochastic motion of nanoparticles was the main mechanism of heat conduction enhancement. They found that, compared with Al_2_O_3_-based nanofluid, the counterpart containing smaller CuO particles had a more significant temperature dependency of thermal conductivity. Agarwal et al. [20] studied the thermal conductivity of kerosene-graphene nanofluids with different specific surface areas (SSA) of GNPs. The results showed that the thermal conductivity enhancement ratio of nanofluids with high SSA particles was higher than that with low SSA particles over a certain concentration range. Yu et al. [21] measured the thermal conductivity of suspensions containing different carbon additives and found that the degree of thermal conductivity increase was closely related to the size and shape of carbon additives.

In addition to considering the effect of the size of nanoparticles on thermal conductivity, many researchers are also interested in the effect of temperature. Das et al. [19] studied the temperature effect of the thermal conductivity enhancement of Al_2_O_3_-water and CuO-water nanofluids. It was found that the thermal conductivity of nanofluids increased by twofold to fourfold with the increase in temperature over the range from 21 °C to 51 °C. Subsequently, Liu et al. [22] investigated the thermal conductivity of graphene-dispersed nanofluids based on ionic liquid 1-hexyl-3-methylimidazolium tetrafluoroborate ([HMIM]BF_4_). Compared to the base fluid, the thermal conductivity of the nanofluid was found to increase by 15.2–22.9% when the temperature ranged from 25 °C to 200 °C. The enhancement in nanofluids relative to base fluids was found to be temperature independent by Timofeeva et al. [23] when they studied water and ethylene glycol nanofluids containing alumina particles. Similarly, Yu et al. [24] prepared a stable ethylene glycol nanofluid containing graphene oxide nanosheets (GONs) and investigated the effect of temperature on thermal conductivity enhancement. The results showed that the enhancement ratio of thermal conductivity was almost constant in the temperature range of 10–60 °C, indicating that the rise in thermal conductivity with increasing temperatures originates from the base fluid rather than from behavior associated with the GONs, which is consistent with the conclusion obtained by Timofeeva et al. [23]. A possible reason was that the effect of the Brownian motion was not obvious due to the large size of GONs.

Based on above literature review, although the influencing factors on the thermal conductivity of nanofluids with GNPs have been systematically studied experimentally, the research on the underlying thermal conductivity mechanism is far from enough, and to the authors’ best knowledge, recent research has focused on water- and glycol-based nanofluids, with very little work having been done on oil-based nanofluids. Therefore, it is necessary to carry out research on the thermal conductivity of nanoparticles in rocket kerosene systems to fill the gap in this research field. Meanwhile, more efforts should be made to further explore the mechanism of the thermal conductivity of nanoparticles to strengthen the understanding and application of this phenomenon.

Although the experiment can directly measure the related new thermal properties, the high experimental cost and limited experimental conditions pose great challenges for the further exploration of graphene at the micro/nano scale. At the same time, in the above experimental studies on thermal conductivity, it can also be found that the increase ratio of the thermal conductivity of nanofluids with the addition of nanoparticles and its temperature dependency are very different among different researchers. These inconsistencies may be due to the different preparation methods of nanofluids [25]. In recent years, with the development of computer technology, the numerical simulation technology applied to study the thermal properties of graphene has become increasingly mature. Molecular dynamics simulation is a powerful computing tool in many numerical simulation software which can provide qualitative and quantitative information in predicting microscopic mechanisms and macroscopic thermodynamic properties. The simulation results obtained by the numerical simulation method have the advantage of good repeatability and avoid manual error.

The molecular dynamics (MD) method has been widely used in the simulation of two-dimensional materials such as graphene [26,27,28,29,30,31,32]. For example, in terms of mechanical properties, Ni et al. [26] studied the anisotropic mechanical properties of graphene sheets by using the MD method. Li et al. [27] reviewed the application of MD simulations in mechanical and tribological properties of carbon nanotubes and graphene sheets. In the pharmaceutical field, Mahdav et al. [28] and Khoshoei et al. [29] studied the adsorption of drugs on graphene nanocarriers using MD simulations to improve drug loading and release properties. In terms of thermal properties, Zhang et al. [30] reviewed the research progress in the MD simulation of novel thermal properties of graphene, such as anisotropic thermal conductivity, decoupled phonon thermal transport, thermal rectification, and interfacial thermal conductivity tunability. In the present paper, we are mainly concerned with the application of graphene in enhancing thermal conductivity. Babaei et al. [33] calculated the thermal conductivity of an n-octadecane/graphene system by using the non-equilibrium molecular dynamics (NEMD) method and found that the thermal conductivity of an n-octadecane/graphene system was enhanced by 52% compared to that of liquid octadecane. Huang et al. [34] studied the enhancement of the thermal conductivity of graphene oxide (GO) and graphene on phase change materials by using the NEMD method. They found that the thermal conductivity of paraffin was 0.373 W/m·K, and that of the composite system by adding graphene and GO was 0.488 W/m·K and 0.506 W/m·K, respectively. Zhang et al. [35] studied the thermal conductivity of the paraffin/EVA/graphene nanocomposites by using the NEMD method and found that the thermal conductivity of the composite system was increased by 16% when the graphene content was 0.7 wt%.

To the best of our knowledge, no MD studies on graphene-rocket kerosene composite systems have been reported so far, and even relevant experiments are scarce. In this paper, the influence of GNPs on the thermal conductivity of rocket kerosene is studied by MD simulations. Firstly, Reverse Non-Equilibrium Molecular Dynamics (RNEMD) and NEMD methods are used to calculate the thermal conductivity of a graphene-rocket kerosene composite system, and their results are compared. Secondly, the effect of the mass fraction and the size of GNPs on the thermal conductivity of the composite system is explored. Finally, the temperature effect on the thermal conductivity of the composite system is investigated. The innovation of this paper is that the effect of graphene size on the thermal conductivity of rocket kerosene is systematically studied, and the thermal conductivity mechanism of graphene in nanofluids is deeply explored. The research in this paper aims to improve the thermal conductivity of rocket kerosene so as to enhance its cooling capacity. Moreover, the mechanism of the thermal conductivity enhancement by adding GNPs is explored, thus providing new ideas for the regenerative cooling of rocket kerosene in the thrust chamber.

## 2. Methods

### 2.1. Construction of the Simulation System

Rocket kerosene is a complex mixture that contains hundreds of components such as alkanes, naphthenes, and aromatic hydrocarbons. In view of the complexity of the composition of rocket kerosene, three representative components from rocket kerosene are selected as the substitute model of rocket kerosene, namely, n-alkanes, monocycloalkanes, and dicycloalkanes, which account for 95.4% of the entire components, and the average carbon numbers of the three components were 13, 13, and 12, respectively. Based on the average carbon numbers of these three types of substances and by investigating related literature, we finally determine the three components of the rocket kerosene model. They are n-tridecane [36], heptylcyclohexane [37], and Decahydro-2,6-dimethylnaphthalene [38]. Each component is added according to its mass percentage. The numbers of molecules and atoms of each component are shown in Table 1.

**Figure 1:**
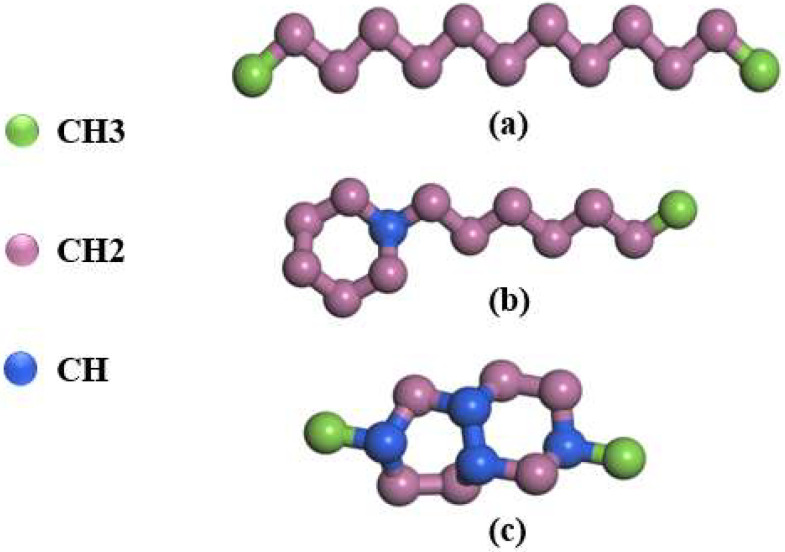
The molecular structure of (**a**) n-tridecane, (**b**) n-heptylcyclohexane, and (**c**) Decahydro-2,6-dimethylnaphthalene in rocket kerosene.

In order to save computational resources, the interaction between alkane molecules is described by the Trappe-UA (Transferable Potentials for Phase Equilibria-United Atom) force field [39] instead of the all-atom model. The idea of the Trappe-UA force field is to unite each carbon and its bonded hydrogen into a single interaction site. Based on this idea, the molecular structure of each component is shown in Figure 1.

**Figure 2:**
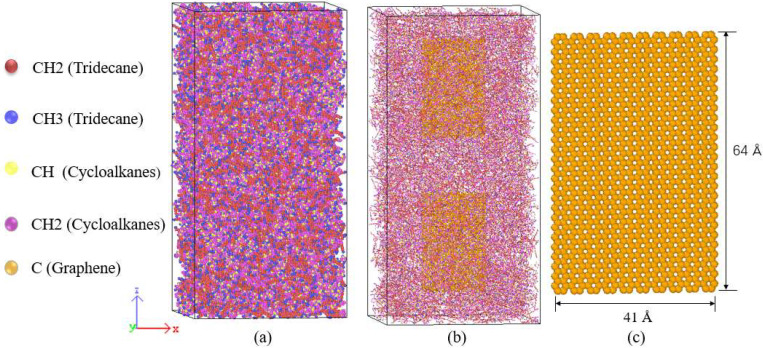
Structures of: (**a**) the rocket kerosene system, (**b**) the graphene-rocket kerosene binary mixture system, and (**c**) GNPs with a size of 41 Å × 64 Å.

In the process of model construction, Packmol [40] is used to generate the initial positions of molecules in the simulation box, and Moltemplate [41] is used to establish the force field and topological relationship. Rocket kerosene molecules are randomly distributed in the simulation box, and GNPs with a mass fraction of 2.28% are added to the rocket kerosene to create a graphene-rocket kerosene composites model. As shown in Figure 2, two pieces of GNPs are initially positioned parallel to the *xz* plane of the simulation system, with a size of 41 Å × 64 Å for each piece. According to the actual density of rocket kerosene, the initial size of the simulated box is determined to be 102 Å × 102 Å × 204 Å. Ovito [42] is used to visualize the simulation results.

### 2.2. Force Field

In view of the complexity of the rocket kerosene model, the use of the all-atom force field is impractical, so we consider using the united-atom force field for our simulations. Previous research results have shown that the Transferable Potential for Phase Equilibria-United Atom (TraPPE-UA) force field can be applied to the simulation of the molecular properties of alkanes [33,43,44]. In addition, the TrapPE-UA force field can accurately reproduce the liquid density among all the force fields with an error of only 1% from the experimental value [44]. Therefore, in this paper, the TraPPE-UA force field is chosen to describe the interaction between rocket kerosene molecules.

The TraPPE-UA field treats the CH*_x_* groups (where 0 < *x* < 4) at the position of the carbon atom as pseudoatoms. By combining H atoms and their associated C atoms into a group, the number of interaction sites is reduced, thus simplifying the parameterization of all atoms and greatly saving computational resources. The nonbonded interactions are described by the simple pairwise-additive Lennard-Jones (LJ) potentials:

(1) $$u(r_{\mathrm{ij}})=\sum_{i,j}4\varepsilon_{ij}\left({\left(\frac{\sigma_{ij}}{r_{ij}}\right)}^{12}-{\left(\frac{\sigma_{ij}}{r_{ij}}\right)}^{6}\right)$$

where *r_ij_* is the distance between atoms *i* and *j,* and *ε_ij_* and *σ_ij_* represent the potential well depth and equilibrium distance between atoms, respectively. The relevant LJ parameters are shown in Table 2. The LJ potential parameters between different pseudoatoms are calculated by the Lorentz-Berthelot mixing rule [45].

(2) $$\sigma_{ij}=\left(\sigma_{ii}+\sigma_{jj}\right)/2$$

(3) $$\varepsilon_{ij}={\left(\varepsilon_{ii}\varepsilon_{jj}\right)}^{1/2}$$

In the TraPPE-UA force field, pseudo-atoms are connected by fixed bond lengths (all bond lengths are 1.54 Å). In order to preserve the complete flexibility of molecules, the bond stretching was considered, and a harmonic potential is used to control bond stretching:

(4) $$u_{stretch}=\frac{1}{2}k_{r}{\left(r-r_{0}\right)}^{2}$$

where *k_r_* is the elastic constant of bond stretching, *r* is the distance between adjacent particles, and *r*_0_ is the equilibrium bond length. Beads separated by two bonds interact by a harmonic bending potential of the form:

(5) $$u_{bend}=\frac{1}{2}k_{\theta }{\left(\theta -\theta_{0}\right)}^{2}$$

where *k_θ_* represents the bending constant, and *θ* and *θ*_0_ represent the bending angle and equilibrium bending angle, respectively. Torsional interactions of pseudo-atoms separated by three bonds are computed using a cosine series.

(6) $$u_{torsion}=c_{0}+c_{1}\left(1+\cos\left(\varphi \right)\right)+c_{2}\left(1-\cos\left(2\varphi \right)\right)+c_{3}\left(1+\cos\left(3\varphi \right)\right)$$

where *φ* is the dihedral angle, and $c_{0}$, $c_{1}$, $c_{2}$, and $c_{3}$ are the Fourier coefficients. All relevant parameters in Equations (4)–(6) are listed in Table 3. For the sake of distinction, pseudoatoms on normal alkanes are represented by CH*_x_* and CH*_y_*, and pseudoatoms on cycloalkanes are represented by CH*_x_*(cyc) and CH*_y_*(cyc). It is worth noting that the interaction parameters of pseudoatoms on the branch chains of cycloalkanes are consistent with those of n-alkanes.

In the simulation of the graphene-rocket kerosene composite system, the adaptive intermolecular reactive empirical bond order (AIREBO) [49] potential is adopted to model the carbon atoms in graphene, since it has been widely used to simulate heat transfer in carbon systems and has produced reliable results [50,51]. The interaction between graphene and the components of rocket kerosene is described by LJ potential [52], in which the parameters are calculated according to the Lorentz-Berthelot mixing rule [45]. The interatomic LJ parameters of graphene are ε=0.00239 ev and σ=3.412 Å, respectively, which are taken from Ref. [53]. The cut-off radius of the LJ potential is set to be 10 Å.

**Table 3:** Bond length, bond bending, and torsional parameters for the TraPPE-UA force field.

**Bond Type**	**Molecule**	***r*_0_ (Å)**		***k_r_* (kcal·mol·Å^−2^)**	
CH*_x_*-CH*_y_* [54]	Alkanes/Branched Alkanes	1.54		899.52	
CH*_x_*(cyc)-CH*_y_*(cyc) [48]	Cycloalkanes	1.54		536	
**Bend type**	**Molecule**	***θ*_0_ (°)**		***k_θ_*/*k*_B_ (K·rad^−2^)**	
CH*_x_*-CH_2_-CH*_y_* [46]	Alkanes/Branched Alkanes	114		62,500	
CH*_x_*(cyc)-CH_2_(cyc)-CH*_y_*(cyc) [47]	Cycloalkanes	114		62,500	
CH*_x_*(cyc)-CH(cyc)-any C [55]	Cycloalkanes	112		62,500	
CH*_x_*(cyc)- CH*_x_*-CH*_y_* [46]	Branched Alkanes	114		62,500	
**Torsion type**	**Molecule**	***C*_0_/*k*_B_ (K)**	***C*_1_/*k*_B_ (K)**	***C*_2_/*k*_B_ (K)**	***C*_3_/*k*_B_ (K)**
CH*_x_*- CH_2_-CH_2_ -CH*_y_* [46]	Alkanes/Branched Alkanes	0	355.03	−68.19	791.32
CH_2_(cyc)-CH_2_(cyc)-CH_2_(cyc)-CH_2_(cyc) [48]	Cycloalkanes	0	355.03	−68.19	791.32
CH*_x_*(cyc)-CH_2_(cyc)-CH(cyc)-any C [48]	Cycloalkanes	−251.06	428.73	−111.85	441.27
CH*_x_*(cyc)-CH(cyc)- CH_2_-CH*_y_* [48]	Cycloalkanes	−251.06	428.73	−111.85	441.27
CH*_x_*(cyc)-CH_2_- CH_2_ -CH*_y_* [46]	Branched Alkanes	0	355.03	−68.19	791.32

### 2.3. Simulation Details

All simulations are performed in large-scale atomic/molecular massively parallel simulators (LAMMPS) [56]. By default, the simulation is performed at 293 K and 0.1 MPa. Firstly, energy minimization is performed using the conjugate gradient algorithm [57]. The Nosé–Hoover thermostat and barostat [58,59] are used to relax the simulation system under the NPT (constant pressure and temperature) ensemble to reach a thermodynamic equilibrium state. The equilibrium stage lasts for 2 ns. Secondly, the Nosé–Hoover thermostat and barostat are removed, and the system is simulated under the NVE ensemble (constant volume without the thermostat and barostat) for another 4 ns to calculate the thermal conductivity. The simulation time step is set to be 1 fs in all simulations. In order to calculate the thermal conductivity more accurately, the RNEMD and NEMD methods are compared.

**A. RNEMD method**

According to Fourier’s law, thermal conductivity can be defined as the proportionality constant of heat flow and the resulting temperature gradient:

(7) $$\lambda =-\frac{J_{z}}{dT/dz}$$

where *J_z_* is the energy transferred through a surface of a given area which is perpendicular to the direction of heat flux at a given time. We assume that the heat is transferred along the *z*-direction. dT/dz is the temperature gradient along the *z*-direction. Generally, there are two widely used methods for the calculation of thermal conductivity, namely, the equilibrium molecular dynamics method (EMD) and the non-equilibrium molecular dynamics method (NEMD). In the EMD method, the thermal conductivity is calculated by the Green Kubo relation [60]. Unfortunately, it is difficult to calculate the transport coefficients of mixtures due to the lack of implicit microscopic expressions for some enthalpy terms in the Green Kubo relation [61,62,63,64]. Therefore, the EMD method is not suitable for calculating the thermal conductivity of our simulation system.

**Figure 3:**
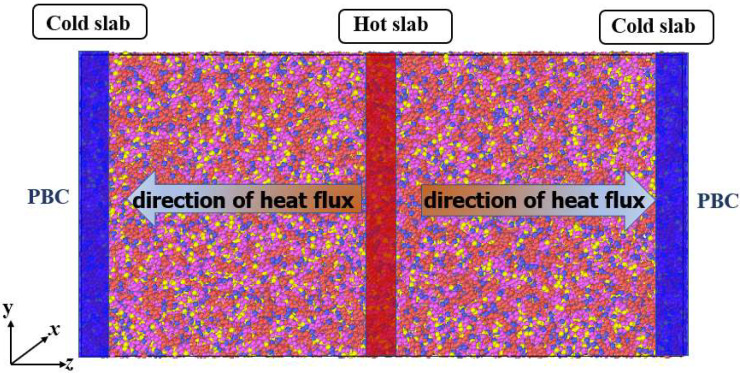
Schematic diagram of the RNEMD method. The first slab is defined as a cold slab (shown in blue) and the middle slab as a hot slab (shown in red). PBC: periodic boundary condition.

Müller-Plathe [65] proposed the RNEMD approach, in which heat flow is applied to the system and the resulting temperature gradient is measured. Following Müller-Plathe’s approach, the simulation box is divided into 20 slabs along the *z* direction, in which the first slab is designated as the cold slab, while the middle slab is designated as the hot slab. The energy exchange is caused by the exchange of kinetic energy between the hottest atom in the cold slab and the coldest atom of the same mass in the hot slab. That means the amount of heat transferred between these two regions is known. The schematic of the thermal conductivity calculation is shown in Figure 3. When the steady state is reached, the generated temperature distribution curve is linearly fitted to obtain the average temperature gradient, and the thermal conductivity is calculated as follows:

(8) $$\lambda =-\frac{\sum_{\mathrm{transfers}}\frac{m}{2}\left(v_{h}^{2}-v_{c}^{2}\right)}{2tA\langle dT/dz\rangle }$$

where *v_h_* and *v_c_* are the velocities of the hottest atom and the coldest atom, respectively; *m* is the atomic mass and *t* is the simulation time; *A* is the cross-sectional area perpendicular to the direction of heat transfer; d*T*/d*z* is the temperature gradient in the *z* direction. The appearance of factor 2 in the denominator is due to the fact that the periodic boundary condition in the *z* direction allows energy to flow in both directions from the hot plate to the cold plate. 〈dT/dz〉 represents the ensemble average of the temperature gradient.

**B** **. NEMD method**

**Figure 4:**
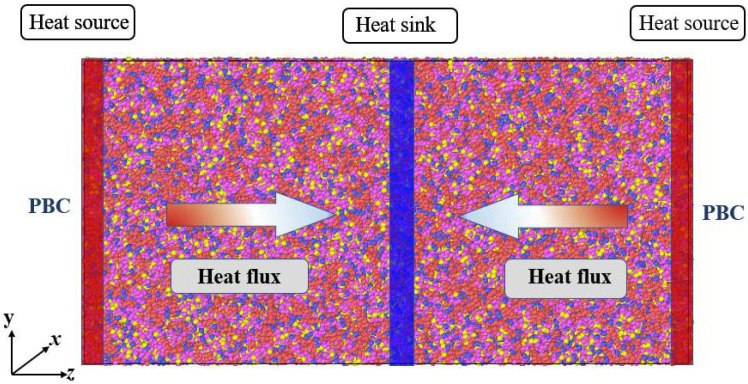
Schematic diagram of the NEMD method. the direction of heat flow is transferred along the z direction, heat flow is applied by adding constant heat energy to the heat source region and subtracting the same amount of energy from the heat sink region. PBC: periodic boundary condition.

Different from the RNEMD method, Jund et al. [66] proposed another method to calculate the thermal conductivity, and the schematic diagram is shown in Figure 4. In this method, the simulation box is divided into 40 layers along the *z* direction. The first layer is defined as the heat source region, while the middle layer is defined as the heat sink region. A certain amount of heat energy is added to the heat source region. Meanwhile, the same amount of heat energy is subtracted from the heat sink region. The magnitude of the heat flux applied to the system is 2.4 × 10^−8^ J/s. Since periodic boundary conditions are used in three directions of the system, the heat flows from the heat source region at both ends of the box to the heat sink area in the middle of the box. The ehex algorithm developed by Wirnsberger et al. [67] was selected to induce heat flux. When the simulation reaches a steady state, the thermal conductivity can be calculated according to Fourier’s law as follows:

(9) $$\kappa \;=\;\frac{dQ/dt}{2A\partial T/\partial z}$$

where d*Q*/d*t* is the rate of heat energy added in the heat source region or subtracted from the heat sink region, A is the cross-sectional area of the simulated box perpendicular to the direction of the applied heat energy, and ∂T/∂z represents the temperature gradients, obtained by averaging the slopes of the temperature profiles on the left and right sides of the simulated box.

## 3. Results and Discussions

### 3.1. Comparison of Simulation Methods

**Figure 5:**
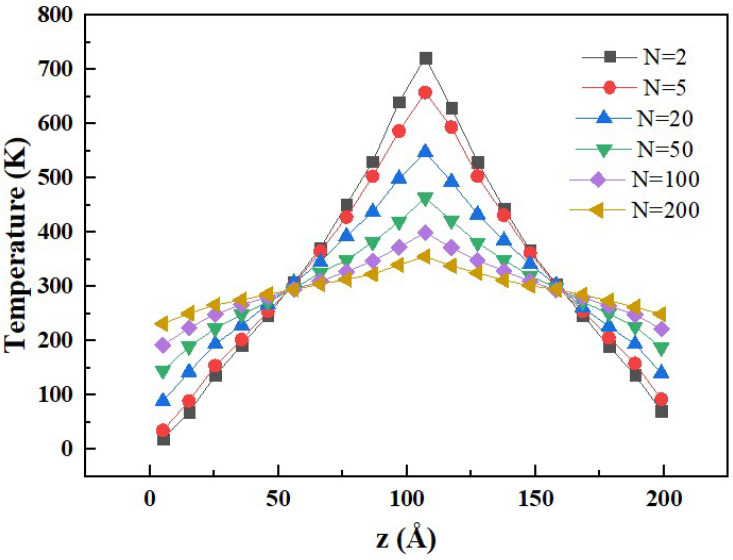
Temperature distribution of the rocket kerosene system along the *z* direction with different exchange intervals *N* of kinetic energy.

In the RNEMD method, the magnitude of the heat flow and the resulting temperature gradient are determined by the exchange interval *N* of kinetic energy. In order to determine the appropriate value of *N*, six different values of *N* are selected to calculate the thermal conductivity. The temperature distribution of the rocket kerosene system along the *z* direction with different values of *N* is shown in Figure 5.

It can be seen from Figure 5 that the larger the value of *N* is, the smaller the temperature difference becomes in the system and the less the simulation system is affected by the temperature difference. Meanwhile, a longer simulation is needed when the temperature difference is smaller. In consideration of both efficiency and accuracy, 200 is selected as an appropriate value of *N*. However, when *N* = 200, it can be observed that the temperature difference within the simulation system is still high—more than 100 K.

**Figure 6:**
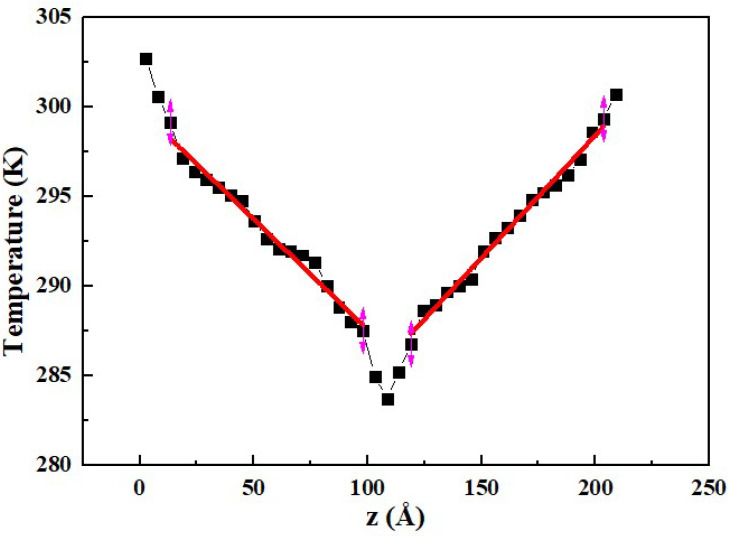
Temperature distribution of the rocket kerosene system along the *z* direction in the NEMD method.

When the NEMD method is used, the temperature distribution of the rocket kerosene system is shown in Figure 6. It can be seen that the temperature difference of the simulated system is less than 25 K. The thermal conductivities calculated by the two simulation methods are listed in Table 4. The thermal conductivities calculated by the MP method and the NEMD method are similar. In view of the influence of the temperature difference within the simulation system on the accuracy of the thermal conductivity calculation, the NEMD method is adopted in the following simulations.

### 3.2. Effect of the Mass Fraction of GNPs on Thermal Conductivity

**Figure 7:**
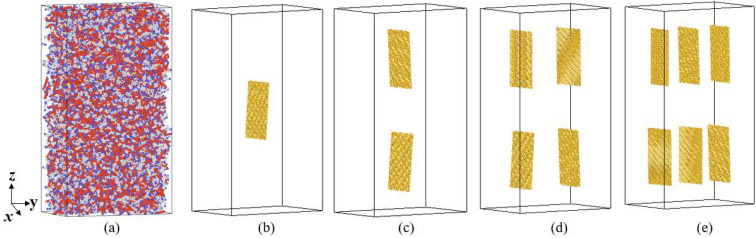
The simulation system of (**a**) the rocket kerosene system and the graphene-rocket kerosene composite systems with different GNPs mass fractions: (**b**) 1.14%, (**c**) 2.27%, (**d**) 4.42%, (**e**) 6.49%.

In order to study the influence of the mass fraction of GNPs on the thermal conductivity of the rocket kerosene system, five models are established for calculation. The model of pure rocket kerosene is used for comparison, and 1, 2, 4, and 6 slices of GNPs are added to the rocket kerosene to construct the graphene-rocket kerosene composite systems with GNP mass fractions of 1.14%, 2.27%, 4.42%, and 6.49%, respectively. The size of a single GNP composed of 1060 carbon atoms is 41.18 × 64 Å^2^. In consideration of the proper arrangement of GNPs, the size of the simulation box is designed to be 80 × 125 × 230 Å^3^. The simulation systems corresponding to the five models are shown in Figure 7. For the convenience of viewing, only GNPs are displayed in the graphene-rocket kerosene composites model.

During the simulation, five independent simulations with different initial velocities of molecules are carried out for each model, and the thermal conductivity is obtained by averaging the results of five simulations. The relevant parameters and calculated thermal conductivity for each model are shown in Table 5.

It can be seen from Table 5 that the thermal conductivity of graphene-rocket kerosene composite systems is generally higher than that of the pure kerosene system, and the thermal conductivity gradually increases as the mass fraction of GNPs increases. The thermal conductivity of the system is enhanced by 4.26%, 7.62%, 15.25%, and 17.83% when the mass fractions are 1.14%, 2.27%, 4.42%, and 6.49%, respectively. According to the idea of the percolation model [13,68,69], the aggregation of nanoparticles during the flow process can form a long chain structure. These chains are interconnected thermal networks that can provide faster heat conduction paths. In addition, as the mass fraction of GNPs increases, the particle–particle distance (mean free path) decreases, resulting in an increase in the frequency of the lattice vibration, thereby enhancing the percolation effect of the heat transfer [70].

### 3.3. Effect of Graphene Size on Thermal Conductivity

**Figure 8:**
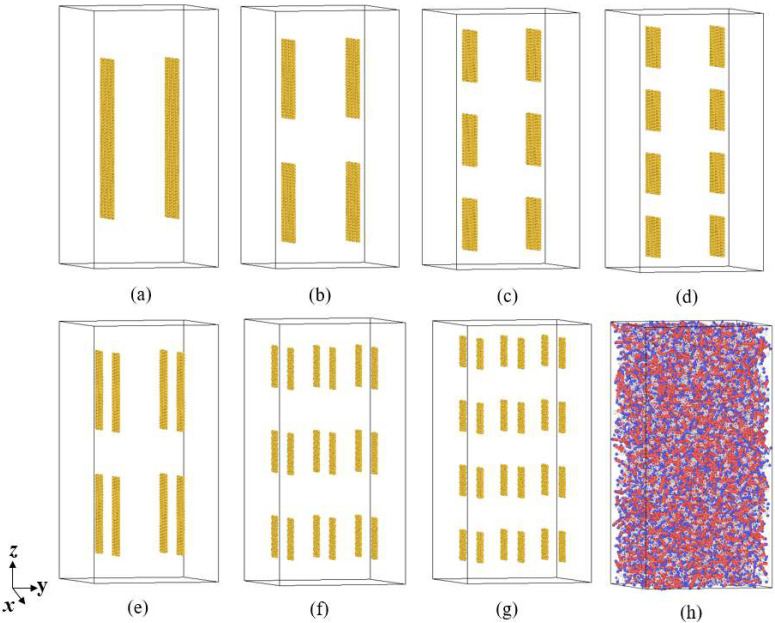
The simulation system of graphene-rocket kerosene composite systems with different GNPs sizes: (**a**) system *a*: GNPs with a size of 41.18 Å × 130.36 Å, (**b**) system *b*: GNPs with a size of 41.18 Å × 64 Å, (**c**) system *c*: GNPs with a size of 41.18 Å × 42.26 Å, (**d**) system *d*: GNPs with a size of 41.18 Å × 31.97 Å, (**e**) system *e*: GNPs with a size of 19.88 Å × 64 Å, (**f**) system *f*: GNPs with a size of 32.66 Å × 17.22 Å, (**g**) system *g*: GNPs with a size of 24.14 Å × 17.22 Å, and (**h**) the rocket kerosene system for comparison.

In order to study the influence of graphene size on the thermal conductivity of rocket kerosene, seven graphene-rocket kerosene composite models are constructed by adding GNPs of different sizes to the rocket kerosene system while keeping the mass fraction of GNPs (4.5%) unchanged. Meanwhile, a rocket kerosene model is constructed for comparison. The distribution of graphene in the graphene-rocket kerosene composite system is shown in Figure 8. All simulation boxes are set to be 106 Å × 106 Å × 112 Å. In order to prevent the aggregation of GNPs, the linear and angular momentums of the GNPs are zeroed during the simulation.

As shown in Figure 8, GNPs are uniformly dispersed in the simulation box. The thermal conductivities calculated from different systems are shown in Table 6. All values of thermal conductivity are averaged from five independent calculations with different initial velocities of molecules. Consistent with the simulation results of Guo et al. [71], we found that the thermal conductivity of the system has a strong dependence on the length of graphene; for example, by comparing the three systems *a*, *b*, and *d* in Table 6, it is found that when the length of GNPs decreases from 130.36 Å to 42.26 Å while keeping the width of GNPs at 41.18 Å, the thermal conductivity of the graphene-rocket kerosene composite system decreases from 20.29% to 8.76%. The same phenomenon can also be observed by comparing systems *f* and *g*. This length dependence of graphene thermal conductivity is caused by the long mean free path of long-wave phonons in graphene [72]. However, with further observations, we find two unexpected results that are inconsistent with the conclusion obtained above. Although the graphene size in system *c* (41 Å × 42 Å) is larger than that in system *d* (41 Å × 32 Å), the thermal conductivity of the former system is lower than that of the latter system. The same unexpected results are found when comparing system *b* and system *e*.

**Figure 9:**
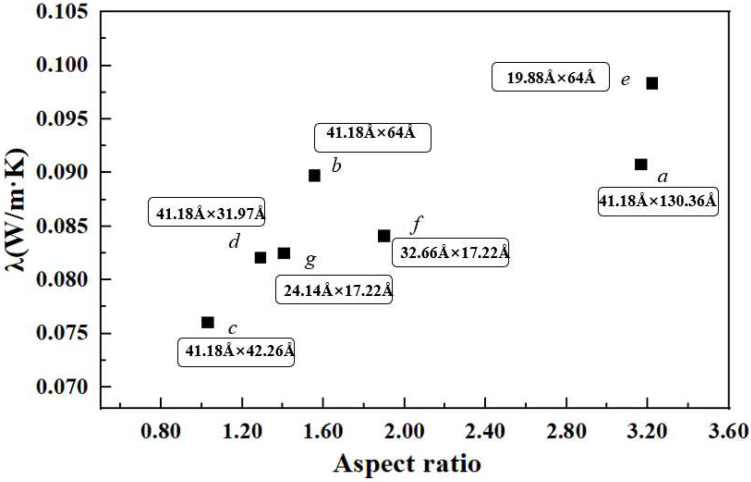
The variation trend of thermal conductivity with the aspect ratio of GNPs at a mass fraction of 4.5%.

In order to determine the reason behind the unexpected results mentioned above, we analyzed the effect of the aspect ratio of GNPs on the thermal conductivity of the composite system. From Figure 9, it can be seen that the thermal conductivity generally increases as the aspect ratio of GNPs increases, indicating that graphene with a higher aspect ratio is more conducive to heat transport. However, we found that the increase in thermal conductivity with the aspect ratio of GNPs is not monotone. Compared to system *b* (41.18 Å × 64.00 Å), system *f* (32.66 Å × 17.22 Å) has a larger aspect ratio of GNPs but a lower thermal conductivity. It is speculated that this may be due to the smaller size of GNPs in system *f*, which results in a shorter effective percolation length. In addition, compared to system *e* (19.88 Å × 64.00Å), system *a* (41.18 Å × 130.36 Å) has a similar aspect ratio of GNPs but a much lower thermal conductivity. This may be caused by the slow Brownian motion of GNPs in system *a* due to their excessive length. Therefore, we believe that the thermal conductivity enhancement mechanism of graphene is jointly controlled by the percolation model and the Brownian motion. GNPs of smaller sizes mainly participate in the Brownian motion to improve the thermal conductivity, while GNPs with larger sizes improve thermal conductivity mainly by the effect of the network chain structure according to the percolation theory. This Brownian motion–percolation hybrid mechanism was first proposed by Gupta et al. [69] through experimental observation, and our study provides evidence for this theory at the molecular level.

### 3.4. Effect of Temperature on Thermal Conductivity

**Figure 10:**
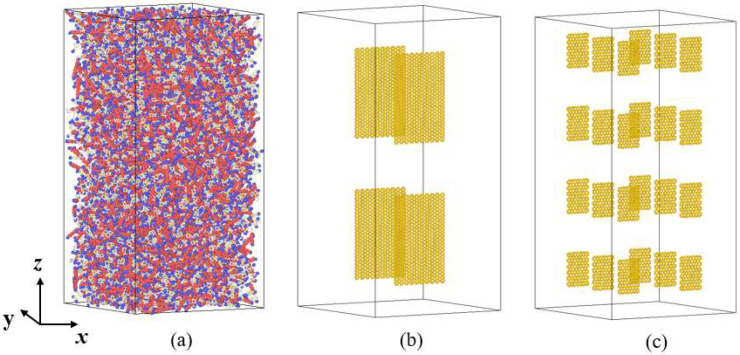
Schematic diagram of (**a**) the rocket kerosene system and the composite system with different GNP sizes: (**b**) system *b*: GNPs with a size of 41.18 Å × 64 Å and (**c**) system *g*: GNPs with a size of 24.14 Å × 17.22 Å.

In order to further explore the heat conduction enhancement mechanism of GNPs, the thermal conductivities are calculated at 293 K, 303 K, 313 K, 323 K, 333 K, and 343 K for three systems: system *b*, system *g*, and the pure rocket kerosene system. The model is shown in Figure 10. During the simulation process, four independent simulations of different initial atomic velocities were carried out for each temperature, and the thermal conductivity was obtained by averaging the results from the four simulations.

**Figure 11:**
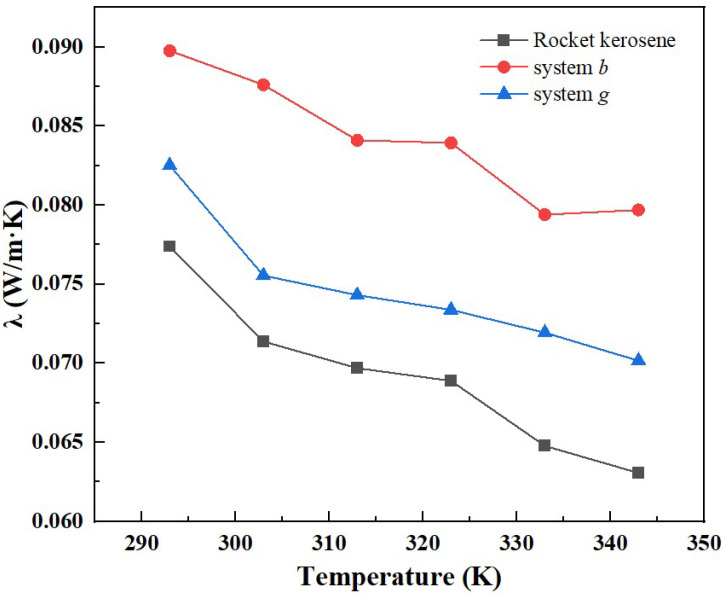
Thermal conductivity of three systems at temperatures varying from 293 K to 343 K.

**Figure 12:**
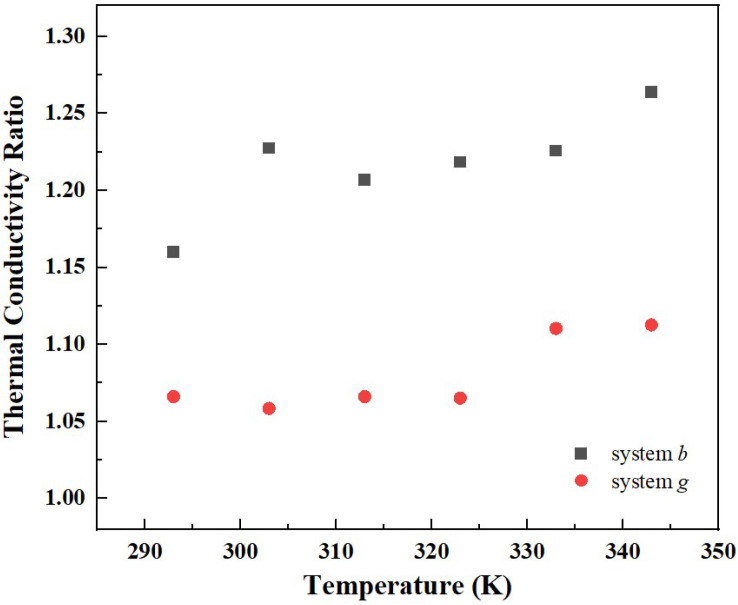
Effect of temperature on the thermal conductivity ratio of the graphene-rocket kerosene composite to rocket kerosene at various particle sizes.

The variation of the thermal conductivity of different systems with temperature is shown in Figure 11. It can be seen that, consistent with the rocket kerosene system, the thermal conductivities of system *b* and system *g* gradually decrease with the increase in temperature. Additionally, the variation of the ratio of the thermal conductivity of composite systems to the rocket kerosene system with temperature is shown in Figure 12. From 293 K to 343 K, for system *b*, the ratio increases from 1.16 to 1.26, and for system *g*, it increases from 1.07 to 1.11. This is consistent with the experimental results obtained by Agarwal et al. [20], who found that the ratio of thermal conductivity increased from 1.23 to 1.30 and from 1.14 to 1.18 for kerosene-graphene nanofluids with a specific surface area (SSA) of 750 m^2^/g and 500 m^2^/g, respectively, when the temperature varied from 20 °C to 70 °C. This temperature effect proves that the Brownian motion of GNPs has non-negligible effects on the thermal conductivity of composite systems.

## 4. Conclusions

In this paper, MD simulations were conducted to study the thermal conductivity of kerosene-based nanofluids containing GNPs. The effects of the mass fraction and the size effect of GNPs as well as temperature on thermal conductivity were investigated, providing a molecular-level understanding of the enhancement mechanism of thermal conductivity by adding GNPs. The main conclusions are as follows:

(1) The thermal conductivity of graphene-rocket kerosene composite systems is higher than that of the pure kerosene system, and it increases as the mass fraction of GNPs increases, which can be related to the enhancement of the percolation effect of heat transfer.
(2) The thermal conductivity increases with the increase in the aspect ratio of GNPs, i.e., graphene with a higher aspect ratio is more conducive to the thermal transport, which indicates that the heat conduction mechanism of graphene in the nanofluid is controlled by both the percolation model and the Brownian motion of GNPs.
(3) The effect of temperature on the thermal conductivity of graphene-rocket kerosene composite systems is found to be consistent with experimental results, i.e., the thermal conductivity decreases with the increase in temperature. Furthermore, the ratio of the thermal conductivity of composite systems to pure rocket kerosene systems increases as the temperature increases, which further proves that the Brownian motion of GNPs has non-negligible effects on the thermal conductivity of composite systems.

The simulation results have important guiding significance for practical applications, and the simulation method in this paper can also be used to explore the influence of other two-dimensional materials on nanofluids [73,74,75,76].

## Figures and Tables

**Table 1:** The numbers of molecules and atoms of each species in the rocket kerosene model.

Species	Components of Rocket Kerosene	Molecular Formula	Molecular Number	Atomic Number
n-alkanes	n-tridecane	C_13_H_28_	1210	15,730
monocycloalkanes	n-heptylcyclohexane	C_13_H_26_	2244	29,172
dicycloalkanes	Decahydro-2,6-dimethylnaphthalene	C_12_H_22_	2827	33,924
	sum	——	6281	78,826

**Table 2:** Lennard-Jones Parameters for the TraPPE-UA force field.

United Atom Type	Molecule	*σ_ii_* (Å)	*ε_ii_*/*k*_B_ (K)
CH_3_ [46]	Alkanes/Branched Alkanes	3.75	98
CH_2_ [46]	Alkanes/Branched Alkanes	3.95	46
CH_2_(cyc) [47]	Cycloalkanes	3.91	52.5
CH(cyc) [48]	Cycloalkanes	4.68	12

**Table 4:** Thermal conductivity calculated by the MP method and the NEMD method.

Method	∆*T* (K)	Model	*λ* (W/m·K)	Enhancement
MP	125	Rocket kerosene	0.0767	——
		Graphene-rocket kerosene	0.0851	10.889%
NEMD	20	Rocket kerosene	0.0770	——
		Graphene-rocket kerosene	0.0869	12.925%

**Table 5:** Model parameters and thermal conductivity under different mass fractions.

Substance	Number	Size	Number of Atoms	Mass Fraction	*λ* (W/m·K)	Enhancement
Rocket kerosene	——	——	78,826	——	0.0774	——
Graphene sheets	1	41.18 Å × 64 Å	1060	1.14%	0.0807	4.26%
	2	41.18 Å × 64 Å	2120	2.27%	0.0833	7.62%
	4	41.18 Å × 64 Å	4240	4.42%	0.0892	15.25%
	6	41.18 Å × 64 Å	6360	6.49%	0.0912	17.83%

**Table 6:** Calculation results of the thermal conductivity of different models.

System	Number of GNPs	Size (*x* × *z*)	Aspect Ratio (*x*/*z*)	*λ* (W/m·K)	Enhancement
Rocket kerosene	——	——	——	0.075	——
*a*	2	41.18 Å × 130.36 Å	3.166	0.091	20.29%
*b*	4	41.18 Å × 64.00 Å	1.554	0.090	18.93%
*c*	6	41.18 Å × 42.26 Å	1.026	0.076	0.78%
*d*	8	41.18 Å × 31.97 Å	1.288	0.082	8.76%
*e*	8	19.88 Å × 64.00 Å	3.219	0.098	30.32%
*f*	18	32.66 Å × 17.22 Å	1.897	0.084	11.47%
*g*	24	24.14 Å × 17.22 Å	1.402	0.083	9.32%

## Data Availability

Not applicable.
